# Supplementary material for: Trimethylamine N-Oxide (TMAO) and Indoxyl Sulfate Concentrations in Patients with Alcohol Use Disorder
Source: Nutrients. 2022 Sep 24;14(19):3964. doi: 10.3390/nu14193964 (PMC9572718; doi:10.3390/nu14193964)
Supplement: Supplementary file 1 [file nutrients-14-03964-s001.zip › Supplemental Tables Coulbault et al.pdf]

Supplemental data – Table S1

Clinical and biological data in AUD patients according to their distribution profile into tertiles of serum TMAO concentrations (tertile 1, [TMAO]<2.55 µM; tertile 2, 2.55 µM≤[TMAO]≤6.76 µM; tertile 3, [TMAO]>6.76 µM]). Statistically significant at p<0.05.

	TMAO concentration			
	**Tertile 1 (n=10)**<br>**[TMAO]<2.55µmol/L**	**Tertile 2 (n=10)**<br>**2.55≤[TMAO]≤6.76µmol/L**	**Tertile 3 (n=10)**<br>**[TMAO]>6.76µmol/L**	
AUDIT	30.2±4.7	26.5±12.7	26.4±8.7	NS
BMI (kg/m²)	22.9±3.6	26.1±5.9	23.2±1.9	NS
Number of meal per day	1.9±1.1	2.1±0.9	2.1±0.9	NS
Cushman's score	5.0±1.8	5.4±2.4	5.2±2.3	NS
Benzodiazepin prescription (days)	6.3±6.2	7.1±5.9	6.8±3.7	NS
BEARNI (/30)	14.0±6.0	12.9±6.0	13.8±6.7	NS
MoCA score (/30)	23.9±4.4	25.0±3.9	24.7±6.8	NS
Length of hospital stay (days)	23.5±7.5	19.9±2.8	17.3±3.6	NS
Total protein (g/L)	67.1±8.3	69.2±6.4	63.0±4.8	NS
Albumin (g/L)	35.9±6.7	38.1±3.8	37.7±3.1	NS
Prealbumin (g/L)	0.25±0.09	0.26±0.09	0.29±0.07	NS
Variation of prealbumin concentration during alcohol withdrawal (g/L)	-0.06±0.09	-0.03±0.08	-0.03±0.05	NS
Variation of albumin concentration during alcohol withdrawal (g/L)	-0.4±7.0	-0.6±4.2	1.8±1.9	NS
γ-glutamyl-transpeptidase (U/L)	806.9±988.3	390.7±933.9	128.9±130.3	NS
Ammonium (µmol/L)	54.9±25.3	42.4±25.3	31.0±13.1	NS
ASAT (U/L)	97.0±72.8	63.0±56.9	49.0±49.6	NS
ALAT (U/L)	44.6±35.6	42.6±42.1	67.7±114.1	NS
ASAT/ALAT ratio	2.5±1.3 $	1.7±0.8	1.3±0.8	p<0.05
Bilirubin (µmol/L)	34.1±21.1	23.8±18.0	17.4±7.0	NS
α2 macroglobulin (g/L)	2.4±0.5 $	2.2±0.5	1.6±0.3	p<0.05
Hyaluronic acid (µg/L)	270.6±536.6	59.6±75.0	34.1±18.4	NS
Score of fibrosis (HEPASCORE)	0.58±0.37	0.49±0.33	0.31±0.17	NS
Serum creatinin (µmoles/L)	51.7±5.3 #$	69.9±12.6	64.1±11.8	p<0.05
Urea (mmoles/L)	2.3±1.0	2.9±0.7	2.8±1.1	NS
Prothrombin time (%)	92.9±12.5	97.2±6.2	98.8±3.4	NS
Haemoglobin (g/dL)	13.0±1.4	14.6±1.3	14.6±0.9	NS
Mean Cellular Volume (µm3)	99.3±7.5	94.9±5.0	99.9±4.3	NS
Platelets (G/L)	149.7±85.8	236.6±66.5	227.7±70.4	NS

#: p<0.05 when compared to tertile 2.

$: p<0.05 when compared to tertile 3.

Supplemental data – Table S2

Clinical and biological data in AUD patients according to their distribution profile into tertiles of serum IS concentrations (tertile 1, [IS]<1,7 µM; tertile 2, 1.7 µM≤ [IS] ≤2.4 µM; tertile 3,[IS]>2.4µM]). Statistically significant at $p<0.05$.

	Indoxyl sulfate concentration			
	**Tertile 1 (n=10)** **[IS]<1.7µmol/L**	**Tertile 2 (n=10)** **1.7≤[IS]≤2.4µmol/L**	**Tertile 3 (n=10)** **[IS]>2.4µmol/L**	p
AUDIT	32.0±4.5	26.9±5.1	24.2±13.8	NS
BMI (kg/m²)	23.4±4.1	25.7±5.2	23.2±3.3	NS
Number of meal per day	1.4±1.0	2.4±0.7	2.3±0.8	p<0.05
Cushman's score	5.9±1.8	4.9±2.4	4.8±2.1	NS
Benzodiazepin prescription (days)	7.9±4.6	5.0±4.9	7.3±6.3	NS
BEARNI (/30)	13.0±4.2	15.2±5.2	12.4±8.3	NS
MoCA score (/30)	25.4±4.4	24.9±6.0	23.3±4.8	NS
Length of hospital stay (days)	23.6±7.2	18.0±4.5	19.1±2.8	NS
Total protein (g/L)	67.0±8.6	66.4±7.7	65.9±4.5	NS
Albumin (g/L)	36.0±6.9	38.3±3.5	37.4±3.0	NS
Prealbumin (g/L)	0.27±0.10	0.29±0.07	0.24±0.06	NS
Variation of prealbumin concentration during alcohol withdrawal (g/L)	-0.08±0.07 $	-0.03±0.05	+0.02±0.07	p<0.05
Variation of albumin concentration during alcohol withdrawal (g/L)	-0.8±6.0	+1.4±2.1	+1.6±5.6	NS
γ-glutamyl-transpeptidase (U/L)	859.8±1261.2	287.1±362.6	179.6±326.2	NS
Ammonium (µmol/L)	45.9±30.1	45.3±26.3	37.3±10.5	NS
ASAT (U/L)	76.2±60.2	69.4±54.0	61.3±73.6	NS
ALAT (U/L)	30.7±17.6	53.9±46.9	68.9±114.4	NS
ASAT/ALAT ratio	2.6±1.3	1.6±0.8	1.4±0.8	p=0.052 Tert.1 vs Tert.3
Bilirubin (µmol/L)	34.6±23.2	21.6±13.7	19.1±9.7	NS
α2 macroglobulin (g/L)	2.2±0.5	1.8±0.6	2.2±0.6	NS
Hyaluronic acid (µg/L)	239.1±521.4	86.3±184.9	38.9±30.7	NS
Score of fibrosis (HEPASCORE)	0.59±0.34	0.34±0.29	0.44±0.29	NS
Serum creatinin (µmoles/L)	56.7±8.8	65.0±15.1	64.0±12.9	NS
Urea (mmoles/L)	2.5±0.9	2.8±1.1	2.6±0.9	NS
Prothrombin time (%)	96.3±8.2	96.5±10.7	96.1±6.6	NS
Haemoglobin (g/dL)	13.5±1.3	14.9±1.4	13.8±1.3	NS
Mean Cellular Volume (µm3)	99.1±6.3	101.1±5.0	94.8±5.8	NS
Platelets (G/L)	167.4±68.7	195.4±66.8	251.2±92.8	p=0.07 Tert.1 vs Tert.3

$: p<0.05 when compared to tertile 3.

Supplemental data - Table S3.A and S3.B

A. Pearson correlations of TMAO concentrations with biological and clinical parameters at the entry.

Variable	r	p
Alpha 2 macroglobulin	-0.5831	0.0007
Glycated haemoglobin	0.6477	0.0008
Natremia	0.4669	0.009
Total protein	-0.4597	0.01
ASAT/ALAT ratio	-0.3992	0.031
Glycemia	0.383	0.037
Hepascore	-0.382	0.037

B. Predictors of TMAO concentration from stepwise multiple linear regression analyses.

All variables with a statistically significant correlation were entered in the regression model, and only predictors of the final solution are presented in the table.

Variable entered	β	(95%CI)	p
Alpha 2 macroglobulin	-0.497	(-0.741;-0.253)	0.000697
ASAT/ALAT ratio	-0.454	(-0.694;-0.214)	0.001353
Natremia	0.423	(0.181;0.665)	0.002502
	adjusted r^2		p
Whole model	0.7059		0.0000127

Supplemental data - Table S4.A and S4.B

A. Pearson correlations of IS concentrations with biological and clinical parameters at the entry.

Variable	r	p
AUDIT	-0.5202	0.0032
Number of meal/day	0.4734	0.0082
Platelets	0.3806	0.038
ASAT/ALAT ratio	-0.3727	0.0465

B. Predictors of IS concentration from stepwise multiple linear regression analyses.

All variables witha statistically significant correlation were entered in the regression model, and only the predictor of the final solution is presented in the table.

Variable entered	β	(95%CI)	p
AUDIT	-0.517	(-0.847;-0.187)	0.004058
	adjusted r²		p
Whole model	0.2404		0.004058

Supplemental data - Table S5.A and S5.B

A. Pearson correlations of variations of serum prealbumin during alcohol withdrawal with others continuous variables.

Variable	r	p
Platelets	0.5878	0.001
Total protein	-0.5843	0.001
GGT (Log)	-0.5028	0.007
ASAT	-0.5024	0.012
Indoxyl sulfate	0.4655	0.014
Potassium	0.4646	0.014
ASAT/ALAT ratio	-0.5024	0.015
Total bilirubin	-0.4566	0.017
Albuminemia	-0.4498	0.018
Hyaluronic acid (Log)	-0.4488	0.019
Cholesterol	-0.4303	0.025
Hepascore	-0.4259	0.027

B. Predictors of variation of serum prealbumin during alcohol withdrawal from stepwise multiple linear regression analyses.

All variables with a statistically significant correlation were entered in the regression model, and only predictors of the final solution are presented in the table.

Variable entered	β	(95%CI)	p
Albuminemia	-0.527	(-0.759;-0.295)	0.00016
Hyaluronic acid (Log)	-0,487	(-0.727;-0.247)	0.000516
Indoxyl sulfate	0.408	(0.170;0.646)	0.002363
	adjusted r^2		p
Whole model	0.668		0.00000433

Supplemental data - Table S6.A and S6.B

A. Pearson correlations of length of hospital stay with others continuous variables.

Variable	r	p
Albuminemia	-0.496	0.005
Glycated haemoglobin	-0.5602	0.0054
TMAO (Log)	-0.4841	0.0067
Prealbuminemia	-0.4331	0.019
Natremia	-0.4226	0.02
Hepascore	0.4163	0.022
Alkaline phosphatase	0.3775	0.0397

B. Predictors of length of hospital stay from stepwise multiple linear regression analyses.

All variables with a statistically significant correlation were entered in the regression model, and only the predictor of the final solution is presented in the table.

Variable entered	β	(95%CI)	p
TMAO (Log)	-0.622	(-0.972;-0.272)	0.001996
	adjusted r^2		p
Whole model	0.3562		0.001996
